# The interplay of genetics and fatty acid metabolism: exploring their impact on metabolic syndrome in Swedish men

**DOI:** 10.1186/s12937-025-01168-8

**Published:** 2025-07-01

**Authors:** Harpa Oskarsdottir, Arnar Palsson, Erla B. Olafsdottir, Vilmantas Giedraitis, Salahuddin Mohammad, Ulf Risérus, Helgi B. Schiöth, Gudrun V. Skuladottir, Jessica Mwinyi

**Affiliations:** 1 Department of Surgical Sciences, Functional Pharmacology and Neuroscience, Uppsala University, Uppsala, Sweden; 2 Department of Physiology, Faculty of Medicine, University of Iceland, Reykjavik, Iceland; 3 Institute of Life and Environmental Sciences, University of Iceland, Reykjavík, Iceland; 4 Department of Public Health and Caring Sciences, Clinical Nutrition and Metabolism, Uppsala University, Uppsala, Sweden

**Keywords:** Single-nucleotide polymorphisms, Metabolic syndrome, Fatty acids, Serum cholesteryl ester, Delta-5-desaturase, High-density lipoprotein cholesterol, Apolipoprotein B

## Abstract

**Background:**

Genetic risk variants for obesity and metabolic syndrome (MetS) have been identified, but their link to relevant metabolic health parameters warrants further attention. This study aimed to investigate the extent to which single-nucleotide polymorphisms (SNPs) associated with obesity are linked to changes in fatty acid (FA) profiles in serum cholesteryl esters, lipid metabolism, and MetS risk.

**Method:**

Data from the Uppsala Longitudinal Study of Adult Men (ULSAM), conducted in men at age 50 (*N* = 1973) and age 70 (*N* = 982), were used to investigate SNPs associated with body mass index (BMI) in genome-wide association studies with metabolic parameters at age 50. The significant SNPs and associated lipid parameters were then used as predictors of MetS over a 20-year follow-up period, at age 70 in binary regression models.

**Results:**

The two genes, the brain-derived neurotrophic factor gene (BDNF) (rs7103411) and the fat mass and obesity-associated gene (FTO) (rs1558902), together with delta-5-desaturase (D5D) activity, 20:5n-3 in serum cholesteryl esters (CE), fasting blood glucose, abdominal skinfold thickness, apolipoprotein-B, and high-density lipoprotein cholesterol (HDL-C) at age 50, significantly predicted the risk of MetS at age 70.

**Conclusion:**

The findings suggest a considerable contribution of the SNPs BDNF rs7103411, FTO rs1558902, and ETV5 rs9816226, along with low D5D activities and serum levels of HDL-C in men at age 50, to the risk for MetS 20 years later.

**Supplementary Information:**

The online version contains supplementary material available at 10.1186/s12937-025-01168-8.

## Background

Obesity and metabolic syndrome (MetS) are complex diseases resulting from interactions between hereditary and environmental factors. They are a major cause of human morbidity and mortality today [1]. Globally, at least 2.8 million people die each year due to being overweight (World Health Organization [WHO], 2021) [2]. The genetic influences on body mass index (BMI) and adiposity traits are estimated to be 30-50%, and this association is more pronounced in individuals with higher BMI [3]. Genome-wide association studies (GWAS) have identified several hundred genetic variants associated with obesity [4]. However, little is known about how genetic predisposition is linked to the metabolism of fatty acids (FAs) and lipids, which are important players in the pathophysiology of obesity and MetS [5, 6, 7].

Disturbances in hepatic FA metabolism are well-documented in patients with MetS, and altered FA profiles and activities of polyunsaturated FA metabolizing desaturases, such as delta-5-desaturase (D5D), have been associated with insulin resistance, diabetes, obesity, and MetS [6, 7]. A study based on data from the Swedish Uppsala Longitudinal Study of Adult Men (ULSAM) showed that the type of FAs in serum cholesteryl esters (CE) was significantly different in middle-aged men who developed MetS at the follow-up investigation 20 years later compared with those who did not [6]. Additionally, it was demonstrated that high stearoyl-CoA desaturase (SCD), high delta-6-desaturase (D6D), and low D5D activity in healthy men at age 50 significantly increased the risk of developing MetS at age 70. The authors suggested that D5D activity might be influenced by genetic factors since the association with D5D activity was not found to relate to lifestyle factors [6]. A recent longitudinal study, which included 148 participants randomly recruited from the Spanish PREDIMED trial, also demonstrated that higher rates of D5D activity are associated with a lower risk of MetS [5]. Genetic variation in the SCD coding gene, SCD1 have been reported to potentially influence individual FA metabolism and, hence, the risk for obesity [8].

Another critical player in the development of obesity and MetS is the reduction of high-density lipoprotein cholesterol (HDL-C) levels, and recent large Finnish population study showed that individuals carrying single-nucleotide polymorphisms (SNPs) linked to low HDL-C levels are susceptible to higher activities in inflammatory pathways [9]. Additionally, a study demonstrated that a genetically beneficial predisposition for high HDL-C levels is weakened by severe obesity [10].

Despite growing recognition of the metabolic dysregulation associated with MetS, the mechanisms underlying disrupted FA metabolism remain poorly understood. While obesity is a key driver of MetS, the exact contributions of obesity-related and MetS-associated SNPs in driving these metabolic disturbances have remained largely unexplored. Crucially, the interplay between genetic susceptibility to higher BMI and alterations in FA profiles or broader lipid metabolism has been insufficiently explored. This knowledge gap limits our understanding of the genetic and metabolic interactions predisposing individuals to metabolic diseases.

In this study, we aimed to determine the predictive value of genetic and metabolic variables in developing MetS over a 20-year period. We examined the association between BMI-related SNPs and changes in key lipid metabolism parameters, including FA desaturation enzyme activity, FA composition in serum cholesteryl esters (CE), triglycerides (TGs), HDL-C, and lipoproteins, in a cohort of men at age 50, and determined the predictive value of identified risk markers in assessing the risk of developing MetS longitudinally at age 70.

## Materials and methods

### Study design and population

The present study was conducted with data from the Uppsala Longitudinal Study of Adult Men (ULSAM) [11]. In 1970, all men born in 1920–1924 residing in Uppsala, Sweden, were invited to participate in the study. At baseline, 2,841 men at age 50 were selected from the register of County Council, of them 82% (*N* = 2,322) accepted to participate. For the present study, baseline participants (age 50 years) and follow-up participants (age 70 years) from the third ULSAM investigation cycle (ULSAM-70) were included. During the intervening 20 years, 422 had died and 219 had moved out of the Uppsala region. Of the 1,681 men invited, 460 did not participate in this follow up. The participation rate was 73% (*N* = 1,221). Both surveys, ULSAM-50 (baseline) and ULSAM-70 (follow-up) were conducted in similar and under standardized conditions (https://www.maelstrom-research.org/dataset/ulsam-50 and https://www.maelstrom-research.org/dataset/ulsam-70).

**Fig. 1:**
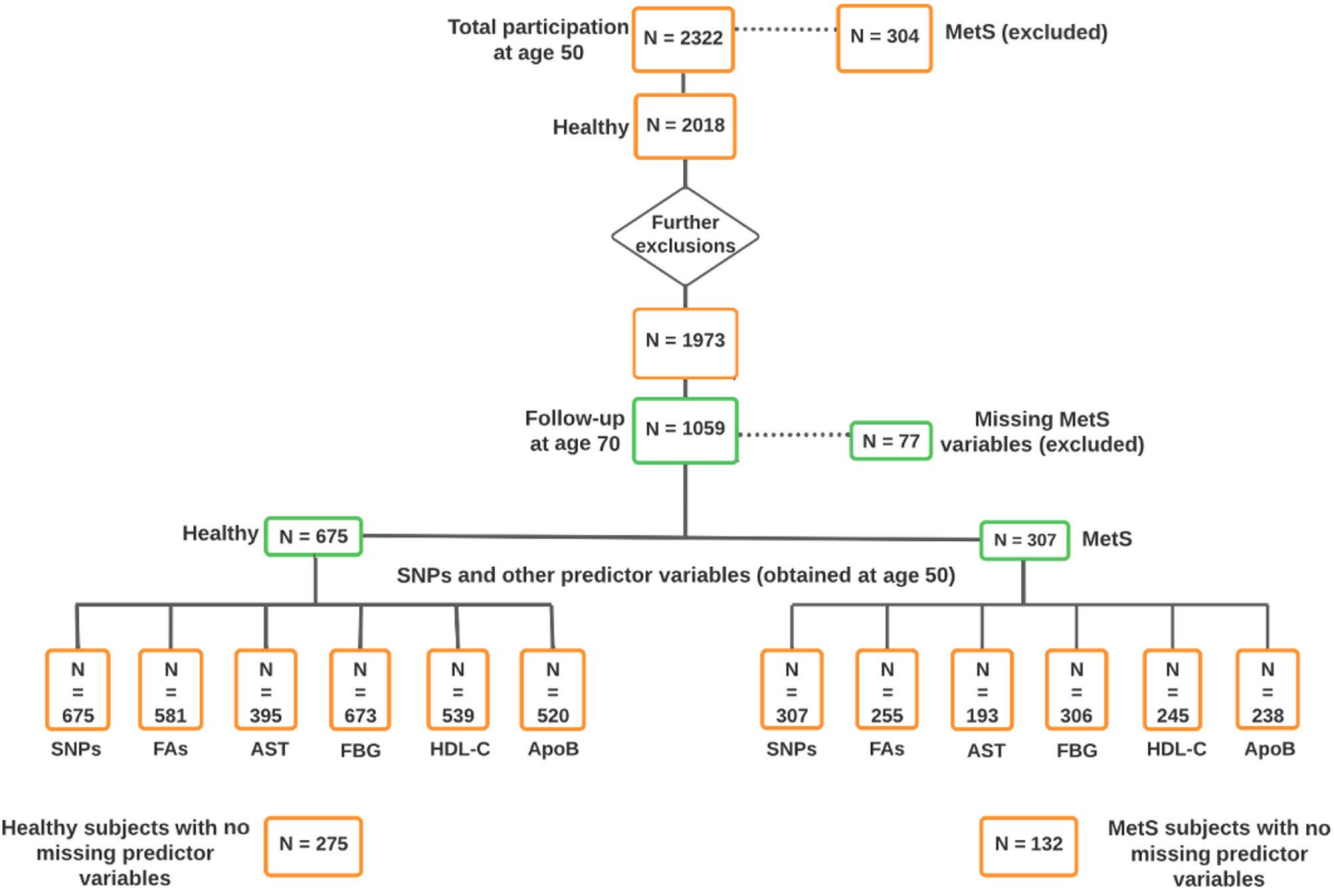
Flowchart of study population. Orange and green boxes indicate individuals with available variables at the age of 50 and 70, respectively. Initially, 304 of 2322 individuals were excluded due to MetS at baseline. Further exclusions included individuals with underweight (*N* = 19) or abundant siblings (*N* = 26). Furthermore to determine MetS, participants with missing one or more variables at age 50 (*N* = 914) and follow-up at age 70 (*N* = 77) were excluded. Finally, 982 participants (675 healthy individuals and 307 individuals with MetS) at the age of 70 were included with baseline and follow-up data. MetS, metabolic syndrome; SNPs, single nucleotide polymorphisms; FAs, fatty acids; AST, abdominal skinfold thickness; FBG, fasting blood glucose; HDL-C, high-density lipoprotein cholesterol; ApoB, apolipoprotein B. Figure created in Lucidchart, www.lucidchart.com

In this study, participants who had MetS at baseline (*N* = 304), participants who were underweight (*N* = 19) and all but one of each sibling group (*N* = 26), and participants who were missing MetS diagnostic variables at age 50 (*N* = 914), and at age 70 (*N* = 77) were excluded. Hence, a total of 982 men were included from the follow-up visit. The flowchart (Fig. 1) schematically shows the number of participants and the variables in the final models. The models (Model 1–8) used the same subjects (*N* = 275 for healthy at age 70, and *N* = 132 for MetS at age 70, total *N* = 407) with complete data, after removing the subjects with missing data (MetS variables age 70 and predictor variables age 50) as shown in the flow chart (Fig. 1). The ULSAM study was approved by the Uppsala University Ethics Committee (Dnr 251/90, approved 1991-08-21) and Regional Ethical Review Authority in Uppsala (Dnr 204:M-382, approved 2004-10-20; Dnr 2013/350, approved 2013-10-23). All participants gave their written informed consent.

### Data collection and preparation

Metabolic and genetic variables from the ULSAM cohort were used in the association analysis and MetS prediction.

#### Metabolic variables

The metabolic variables included FAs in CE and their desaturases, abdominal skinfold thickness (AST), fasting blood glucose (FBG), TG, cholesterol, low-density lipoprotein cholesterol (LDL-C), HDL-C, apolipoprotein B (apoB), systolic blood pressure (SBP), and diastolic blood pressure (DBP). Data collection and investigations were performed under standardised conditions and have been described in detail previously by Byberg et al. [12] and Vessby et al. [13]. The investigations comprised a medical questionnaire and interview, along with blood sampling, anthropometric measurements, and blood pressure assessment. Blood samples were collected after an overnight fast to measure triglycerides, lipoproteins, serum cholesterol, serum cholesteryl ester fatty acids, and blood glucose levels. Blood glucose was analyzed using a spectrophotometer with the glucose oxidase method. BMI was calculated as weight (kg) divided by height (m) squared. MetS was defined as having three or more of the following: Large waist circumference (≥ 102 cm), high TG and/or lipid medication (≥ 1.7 mmol/L), low HDL-C (< 1.04 mmol/L), elevated DBP (≥ 85 mmHg) and/or elevated SBP (≥ 130 mmHg) and/or anti-hypertensive medication, high FBG (≥ 5.6 mmol/L) [14]. Same criteria were used to define diagnoses at age 70, with the addition of lipid lowering medication and diabetes medication as proxies for values of HDL-C (< 1.04 mmol/L), TG (≥ 1.7 mmol/L), and FBG (≥ 5.6 mmol/L).

Methods used for serum CE extraction and FA analysis has been previously described [6, 15]. Additional details on the laboratory methods used in ULSAM are available on the ULSAM study website (https://www.uu.se/forskning/ulsam). Activity indices of the enzymes involved in FA metabolism were determined by calculating the ratios of the product/precursor FAs derived from serum CE. Specifically, the activity indices of SCD-16 (16:1n-7/16:0), SCD-18 (18:1n-9/18:0), D6D (18:3n-6/18:2n-6), and D5D (20:4n-6/20:3n-6) were calculated and considered in subsequent statistical analyses.

#### Genetic variables

Genotyping was carried out at the SNP & SEQ technology platform at Uppsala University (https://snpseq.medsci.uu.se). SNPs were genotyped using a high throughput microarray technology, captured by either the Illumina 2.5 M or Cardio-Metabo chip (combined dataset). SNPs on the *SCD1* gene (rs10883463, rs7849, rs50384) [8], as well as SNPs significantly associated with BMI reported in GWAS [16] were selected. SNPs present on the Illumina 2.5 M or Cardio-Metabo chip were used for further analyses. Thirty-five SNPs, including proxies, within or close to 32 genes spread over 16 chromosomes were chosen for the study (Table 1).

**Table 1:** List of SNPs selected for the presented study

Chr. No.	SNP ID	Gene
1	rs2815752	*NEGR1*
1	rs1514175	*TNNI3K*
1	rs1555543	*PTBP2*
1	rs543874	*SEC16B*
2	rs2867125	*TMEM18*
2	rs713586*	*RBJ*
2	rs887912	*FANCL*
2	rs2890652*	*LRP1B*
3	rs13078807	*CADM2*
3	rs9816226	*ETV5*
4	rs10938397	*GNPDA2*
4	rs13107325	*SLC39A8*
5	rs2112347	*FLJ35779*
5	rs4836133*	*ZNF608*
6	rs206936	*NUDT3*
6	rs987237	*TFAP2B*
9	rs10968576	*LRRN6C*
10	rs10883463	*SCD1*
10	rs7849	*SCD1*
10	rs508384	*SCD1*
11	rs4929949	*RPL27A*
11	rs10767664*	*BDNF*
11	rs3817334	*MTCH2*
12	rs7138803	*FAIM2*
13	rs4771122	*MTIF3*
14	rs11847697	*PRKD1*
14	rs10150332*	*NRXN3*
15	rs2241423	*MAP2K5*
16	rs12444979	*GPRC5B*
16	rs7359397	*SH2B1*
16	rs1558902	*FTO*
18	rs571312	*MC4R*
19	rs29941	*KCTD15*
19	rs2287019	*QPCTL*
19	rs3810291	*TMEM160*

Chr, Chromosome; SNP ID, Single Nucleotide Polymorphism Identification number *Original SNP missing from the data

Quality control and association analysis were carried out using Plink (version 2.0; Free Software Foundation Inc., Boston, MA, USA; https://zzz.bwh.harvard.edu/plink). Linkage disequilibrium (LD) and haplotypes were estimated using the software program Haploview. Chromosomal position data was based on GRCh37.p13. SNPs with minor allele frequency (MAF) > 1% and Hardy-Weinberg equilibrium (HWE) with *P* < 0.001 were included, and only one SNP in each LD was selected in the final models (Figure S1). Proxy SNPs (Table S1) were investigated, for SNPs not available in the original data or for those not fulfilling the chosen criteria for HWE or MAF, and had to correlate with r^2^ > 0.8 with the original SNPs (Table 1). Individuals with a genotyping call rate < 95% were removed. In familial data, only one individual of siblings, either formally known from records or suspected from genetic analysis, was considered in the analysis. In this case, individuals with the most complete data, both genotype and phenotype, were chosen for further downstream analysis or randomly selected in case equal amounts of data were available.

### Statistical analysis

Intra-individual and inter-individual comparison of metabolic variables were performed by using Wilcoxon [17] and Kruskal-Wallis [18] test. MetS risk at the age of 70 were explored using genetic and metabolic variables previously shown to be associated with obesity and/or MetS, and were considered in the statistical analysis. In the first step, association analyses between SNPs and FAs in serum CE and desaturase enzymes at ages 50 and 70 were performed using Plink 2.0 (see www.cog-genomics.org/plink/2.0/) [19]. Results were corrected using Benjamini-Hochberg false discovery rates (FDR) [20] with a threshold < 0.2 (Table S2a). Non-normally distributed variables, i.e., FAs and AST, were log-transformed in the case of positively skewed variables or squared in the case of negatively skewed variables. Outliers were matched to the nearest value. SNPs were coded as 0 for non-carriers, 1 for heterozygotes, and 2 for homozygotes of the minor allele, assuming an additive effect. Only SNPs that were associated with other variables of interest were considered in the binomial logistic regression models.

Binary logistic regression analysis was carried out to estimate the risk of having MetS at age 70, in relation to genotypes of the SNPs of interest and variables at age 50 as predictor variables by creating eight statistical models (Model 1–8). These variables were the ‘Metabolic variables’ and ‘Genetic variables’ (Data preparation section above). MetS status (as defined in Data preparation section above) after 20 years was the prediction target of dichotomous classifiers (0 for no; 1 for yes). In *model 1*, the association between MetS at age 70 and relevant BMI and SCD1-related SNPs, previously shown to be associated with FA metabolism was analysed. In *model 2*, D5D levels at age 50 and their related SNPs were considered in association analyses with MetS at age 70. The subsequent models examined associations between various factors at age 50 and MetS at age 70. *Model 3* focused on the activity indices of FA-metabolizing enzymes, while *model 4* analyzed FA levels in serum CE. *Model 5* investigated indicators of belly fat and glucose imbalance, specifically AST and glucose. *Model 6* explored serum lipids and lipoproteins, and *model 7* assessed blood pressure. The backward elimination method was used. Only significant predictors (P < 0.05) of all variables from model 1 to 7 were used in the final additive model (*Model 8*). Variables with high correlations (R > 0.7) were not included. Statistical analysis was carried out using the open-source R statistical software (see http://www.R-project.org/).

## Results

### Baseline characteristics

Of the 982 individuals enrolled in the follow up study, 307 had MetS at age 70, but 675 did not meet MetS criteria and considered as healthy (neither at age 50 nor 70) (Fig. 1). The baseline characteristics of the individuals with MetS at ages 50 and 70 compared with those without MetS is shown in Table 2. To avoid any potential selection bias, and to determine the representativeness of the longitudinal cohort, the baseline characteristics of the participants from ULSAM-50, but not available during ULSAM-70 were evaluated. The findings were in the same direction as the study participants and mentioned in the supplementary material ‘Table S3’.

**Table 2:** Baseline characteristics of individuals at ages 50 and 70 in the ULSAM cohort stratified according to MetS status at age 70

	No MetS, age 70 (*n* = 675)		MetS, Age 70 (*n* = 307)		Intra-individual differences #		Inter-individual differences ##	
	Age 50	Age 70	Age 50	Age 70	No MetS, Age 70	MetS, Age 70	Age 50	Age 70
BMI (Kg/m^2^)	23.9 (36, 19)	25 (36, 18.6)	26 (38, 19)	28 (46, 20)	***	***	***	***
AST (mm)	16.2 (50, 3.8)	N/A	24 (50, 6)	N/A	N/A	N/A	**	N/A
WC (cm)	84 (109, 69)	92 (118, 51)	88 (113, 74)	101 (137, 76)	***	**	*	***
SBP (mmHg)	125 (210, 100)	143 (207, 102)	130 (210, 100)	150 (201, 100)	***	***	*	**
DBP (mmHg)	80 (125, 50)	82 (111, 57)	80 (140, 60)	86 (115, 62)	**	ns	*	*
FBG (mmol/L)	4.8 (8, 3)	5.3 (18, 3.6)	5.0 (10.8, 3.9)	5.9 (16, 4.3)	***	***	*	***
TC (mmol/L)	6.5 (15.5, 3.8)	5.8 (8.7, 2.4)	7.1 (15, 3.4)	5.9 (9.7, 2.9)	***	***	**	ns
HDL-C (mmol/L)	1.4 (3, 0.4)	1.3 (3.1, 0.5)	1.3 (2.7, 0.3)	1.1 (3, 0.6)	**	**	**	***
LDL-C (mmol/L)	4.9 (13.5, 2.4)	3.8 (6.5, 1.3)	5.6 (13.4, 2.7)	3.9 (6.9, 1.6)	***	***	**	ns
Non-HDL-C (mmol/L)	5.2 (14, 2.5)	4.4 (7.5, 1.6)	5.9 (13.8, 2.8)	4.8 (8.5, 2)	***	***	**	*
TG (mmol/L)	1.4 (5.5, 0.7)	1.1 (4.1, 0.3)	1.8 (10.2, 0.6)	1.7 (5.9, 0.5)	***	*	***	***
ApoB (g/L)	1.1 (2.3, 0.5)	N/A	1.3 (2.5, 0.8)	N/A	N/A	N/A	**	N/A

Data presented as Median (range). BMI, body mass index; AST, abdominal skinfold thickness; WC, waist circumference; SBP, systolic blood pressure; SBP, diastolic blood pressure; FBG, fasting blood glucose; TC, total cholesterol; TG, triglyceride; ApoB, apolipoprotein B; ns, not significant; N/A, not available

*P < 0.001; **P < 1 × 10^− 7^; ***P < 1 × 10^− 15^

#: Intra-individual differences stratified according to MetS at age 70 analysed by Wilcoxon test

##: Inter-individual differences stratified according to MetS at age 70 analysed by Kruskal Wallis test

The median (range) levels were significantly higher for SBP at 130 (210, 100) vs. 125 (210, 100) mmHg (*P* < 0.001) and 150 (201, 100) vs. 143 (207, 102) mmHg (*P* < 1 × 10⁻⁷). Similarly, DBP was elevated at 80 (140, 60) vs. 80 (125, 50) mmHg (*P* < 0.001) and 86 (115, 62) vs. 82 (111, 57) mmHg (*P* < 0.001). FBG levels were also higher at 5.0 (10.8, 3.9) vs. 4.8 (8, 3) mmol/L (*P* < 0.001) and 5.9 (16, 4.3) vs. 5.3 (18, 3.6) mmol/L (*P* < 1 × 10⁻¹⁵). Likewise, TG levels increased at 1.8 (10.2, 0.6) vs. 1.4 (5.5, 0.7) mmol/L (*P* < 1 × 10⁻¹⁵) and 1.7 (5.9, 0.5) vs. 1.1 (4.1, 0.3) mmol/L (*P* < 1 × 10⁻¹⁵). Compared with the individuals without MetS, the individuals with MetS had significantly lower median (range) levels of HDL-C at 1.3 (2.7, 0.3) vs. 1.4 (3, 0.4) mmol/L (*P* < 1 × 10^− 7^), and 1.1 (3, 0.6) vs. 1.3 (3.1, 0.5) mmol/L (*P* < 1 × 10^− 15^) at age 50 and 70, respectively.

**Table 3:** Association analyses between BMI-related SNPs and lipid associated variables at age 50

Chr/Gene (SNP ID) (*n* of 0/1/2)	SCD16 β (*P*-value)	D5D β (*P*-value)	Elongase β (*P*-value)	16:1n-7 β (*P*-value)	22:6n-3 β (*P*-value)	HDL-C β (*P*-value)
1/PTBP2 (rs1555543) (301/460/154)	0.011 (0.01)	ns	ns	0.15 (0.004)	ns	ns
2/FANCL (rs887912) (479/373/63)	0.014 (0.003)	ns	-0.21 (0.004)	0.16 (0.008)	ns	ns
3/ETV5 (rs9816226) (635/269/11)	ns	ns	ns	ns	ns	-0.078 (0.002)
4/GNPDA2 (rs10938397) (313/435/167)	ns	ns	0.19 (0.004)	ns	ns	ns
4/SLC39A8 (rs13107325) (846/67/2)	ns	-0.58 (0.02)	ns	ns	ns	ns
5/FLJ35779 (rs2112347) (368/411/136)	ns	-0.25 (0.01)	ns	ns	ns	ns
10/SCD1 (rs10883463) (788/122/5)	ns	0.67 (0.0002)	ns	ns	ns	ns
10/SCD1 (rs508384) (620/273/22)	ns	0.25 (0.05)	ns	ns	ns	ns
10/SCD1 (rs7849) (616/273/22)	ns	0.25 (0.05)	ns	ns	ns	ns
11/BDNF (rs7103411) (563/308/44)	ns	0.25 (0.03)	ns	ns	ns	ns
13/MTIF3 (rs1006353) (509/342/64)	-0.012 (0.01)	ns	0.19 (0.009)	-0.16 (0.008)	0.029 (0.004)	ns
16/FTO (rs1558902) (336/430/144)	ns	-0.21 (0.03)	ns	ns	ns	ns
19/TMEM160 (rs3810291) (336/430/144)	ns	-0.29 (0.003)	ns	ns	ns	ns

Chr, chromosome; SNP, single nucleotide polymorphism; ID, identification number; SCD-16, steroyl-CoA desaturase (16:1n-7/16:0); D5D, delta-5-desaturase ((20:4n-6/20:3n-6); elongase (18:1n-9/16:1n-7); HDL-C, high-density lipopoprotein cholesterol. Beta values are with respect to the minor allele. 0 are homozygotes for the major allele, 1 are heterozygotes, and 2 are homozyogotes for the minor allele. ns, not significant; β, beta value. Details in supplementary material Table S2a

### Association of BMI-related SNPs and lipid associated variables at age 50

Several BMI-related SNPs were associated with one or more enzymes involved in FA metabolism and lipoproteins at age 50 (SCD-16, D5D, elongase, 16:1n-7, 22:6n-3, and HDL-C), as shown in Table 3. Notably, most SNPs were associated with D5D at age 50. SNP rs9816226 of *ETV5* was inversely associated with HDL-C.

**Table 4:** Binary regression models predicting MetS at age 70 based on snps, clinical and laboratory variables important for metabolic health at age 50

Model	SNP and parameter type considered at age 50	Specific variables considered for each model	Variables that reached significance (*P* < 0.05)
Model 1	SNPs, associated with BMI in GWAS or on the SCD1 gene, that were associated with variables of the ULSAM cohort in the SNP association analysis	PTBP2, FANCL, ETV5, GNPDA2, SLC39A8, FLJ35779, SCD1, BDNF, MTIF3, FTO, TMEM160 *	*ETV5* rs9816226 SLC39A8 rs13107325 *FTO* rs1558902
Model 2	D5D at age 50 and SNPs associated with D5D	SLC39A8 rs13107325, FLJ35779 rs2112347, SCD1 rs10883463, BDNF rs7103411, FTO rs1558902, TMEM160 rs3810291	SLC39A8 rs13107325 *BDNF* rs7103411^+^ *FTO* rs1558902^+^ D5D
Model 3	Activity indices of enzymes involved in FAs	SCD-16, SCD-18 D6D, D5D, elongase	SCD-18, D6D, D5D
Model 4	FA values derived from serum CE	16:0, 16:1n-7, 18:0, 18:1n-9, 18:2n-6, 18:3n-6, 20:3n-6, 20:4n- 6, 18:3n-3, 20:5n-3, 22:6n-3	16:1n-7, 18:0, 18:3n-3, 20:3n-6, 20:5n-3
Model 5	Values reflecting abdominal obesity and insulin resistance	AST, FBG	AST, FBG
Model 6	Serum total cholesterol, TG and lipoproteins	Total cholesterol**, TG, non-HDL-C, HDL-C, LDL-C**, ApoB	TG, HDL-C, ApoB
Model 7	Blood pressure	SBP, DBP, antihypertensive medication	DBP
Model 8	Combined model using SNPs and variables at age 50 from models 2–7 as risk factors for MetS at age 70	***	*BDNF* rs7103411, *FTO* rs1558902, D5D, 20:5n-3, AST, FBG, HDL-C, ApoB

Binary regression models were made using SNPs and variables reflecting metabolic health at age 50. The outcome variable of each model was MetS status at age 70. All models included only the subjects (*N* = 407) with complete data (no missing variables) (Fig. 1). MetS, Metabolic syndrome; SNPs, single nucleotide polymorphisms; BMI, body mass index; SCD1, steroyl-CoA desaturase; D5D, delta-5 desaturase; FAs, fatty acids; CE, cholesteryl ester; AST, abdominal skinfold thickness; FBG, fasting blood glucose; TG, triglycerides; HDL-C, high-density lipoprotein cholesterol; LDL-C, light-density lipoprotein; ApoB, apolipoprotein B. *See Figure S1, ** Only significant predictors (*P* < 0.05) were used in the final version of the serum lipids and lipoproteins model. Total cholesterol and LDL-C were removed because of high inter-correlations (*R* > 0.7). *** D6D, 16:1n-7, 18:0, 20:3n-6 were excluded because of inter-correlations

### Link between SNPs and risk of MetS at the age of 70 (Model 1)

The associations of the significant SNPs, ETV5 rs9816226, SLC39A8 rs1317325, and FTO rs1558902 (Table 4, Model 1), showed an increased risk of MetS at age 70 (Table 5, Model 1). The genetic effects at *ETV5* rs9816226 and *FTO* rs1558902 resembled dominant inheritance, but the SLC39A8 rs1317325 indicated an increased risk in heterozygotes.

**Table 5:** Results from models; with SNP effects (Model 1), or SNP and D5D influence (Model 2) at age 50, activity indices of SCD-18, D6D, and D5D, FAs in serum cholesteryl esters, FBG and AST, serum lipid lipoprotein, and DBP (Model 3–7) as predictors for MetS at age 70

	Variables	OR	95% CI	*P*-value
**Model 1**	*ETV5* rs9816226			
	*1*	0.86	0.62, 1.17	0.3
	*2*	4.89	1.37, 22.8	0.02
	*ETV5* rs9816226			
	*1*	0.86	0.62, 1.17	0.3
	*2*	4.89	1.37, 22.8	0.02
	*SLC39A8* rs13107325			
	*1*	2.18	1.31, 3.63	0.003
	*2*	0.00		> 0.9
	*FTO* rs1558902			
	*1*	1.14	0.83, 1.57	0.4
	*2*	1.55	1.02, 2.35	0.04
**Model 2**	*SLC39A8* rs13107325			
	*1*	2.00	1.15, 3.45	0.01
	*2*	0.00		> 0.9
	*BDNF* rs7103411			
	*1*	1.23	0.88, 1.72	0.2
	*2*	1.90	0.91, 3.89	0.08
	*FTO* rs1558902			
	*1*	1.22	0.86, 1.72	0.3
	*2*	1.48	0.93, 2.34	0.09
	D5D	0.11	0.02, 0.49	0.004
**Model 3**	SCD-18	0.22	0.05, 0.99	< 0.05
	D6D	5.58	2.54, 12.5	< 0.001
	D5D	0.18	0.04, 0.77	0.02
**Model 4**	16:1n-7	6.00	1.50, 24.3	0.01
	18:0	6.74	1.40, 32.8	0.02
	18:3n-3	0.22	0.05, 1.01	0.05
	20:3n-6	24.3	4.79, 128	< 0.001
	20:5n-3	2.91	1.24, 6.87	0.01
**Model 5**	FBG	1.54	1.11, 2.18	0.01
	AST	28.3	11.2, 74.7	< 0.001
**Model 6**	TG	1.69	1.26, 2.30	< 0.001
	HDL-C	0.44	2.25, 0.76	0.004
	ApoB	5.87	2.78, 12.7	< 0.001
**Model 7**	DBP	1.03	1.02, 1.05	< 0.001

OR, odds ratio; CI, confidence intervals; D5D, delta-5-desaturase; SCD-18, stearoyl-CoA desaturase (18:1n-9/18:0); D6D, delta-6 desaturase; FBG, fasting blood glucose; AST, abdominal skinfold thickness; TG, triglycerides; HDL-C, high-density lipoprotein cholesterol; ApoB, apolipoprotein B; DBP, diastolic blood pressure. The SNPs were coded as 1 for heterozygotes, 2 for homozygotes for minor alleles, and reference were 0 for non-carriers (not shown). D5D, AST, and all FA predictor variables in the models were log 10-transformed before the regression analysis. All the binary logistic models used variables as predictors, and MetS at age 70 as the outcome variable among the subjects (*N* = 407) with complete data (no missing variables) (Fig. 1)

### 3.4. Link between BMI-SNPs related to D5D and risk of MetS at the age of 70 (Model 2)

Among the significant D5D-related SNPs and D5D variables at age 50 (Table 4, Model 2), both SLC39A8 rs13107325 and D5D were statistically significant to predict risk of MetS at age 70 (Table 5, Model 2). SLC39A8 rs13107325 heterozygotes (OR = 2.00, *P* = 0.01) along with low levels of D5D (OR = 0.11, *P* = 0.004) significantly increased the risk of MetS at age 70.

### 3.5. *Link between metabolic variables and risk of MetS at the age of 70 (Model 3–7)*

FA related enzyme activities, lipid profiles, and metabolic factors were explored at age 50 (Table 4, Model 3–7, Figure S2-S3) for suitability as predictor for the risk of MetS at age 70 (Table 5, Model 3–7).

**Table 6:** BMI-related snps, clinical metabolic risk parameters, and FA and lipoprotein related variables at age 50 as predictors for MetS at age 70 (Model 8)

Variable	OR	95% CI	*P*-value
*BDNF* rs7103411			
*1*	1.95	1.15, 3.35	0.01
*2*	2.33	0.74, 7.28	0.1
*FTO* rs1558902			
*1*	1.18	0.68, 2.06	0.6
*2*	3.07	1.47, 6.52	0.003
D5D	0.05	0.00, 0.61	0.02
20:5n-3	15.5	3.60, 70.6	< 0.001
FBG	1.84	1.17, 2.98	0.01
AST	17.7	5.06, 66.4	< 0.001
HDL-C	0.16	0.06, 0.39	< 0.001
ApoB	10.2	3.80, 29.1	< 0.001

OR, odds ratio; CI, confidence interval; D5D, delta-5 desaturase; FBG, fasting blood glucose; AST, abdominal skinfold thickness; HDL-C, high-density lipoprotein cholesterol; apoB, apolipoprotein B

The SNPs were coded as 1 for heterozygotes, 2 for homozygotes for minor alleles, and reference were 0 for non-carriers (not shown). D5D, 20:5n-3, and AST levels were log10-transformed before the regression. Binary logistic model using SNPs and metabolic variables at age 50 as predictors, and MetS at age 70 as the outcome variable among the subjects (*N* = 407) with complete data (no missing variables) (Fig. 1)

*In model 3*, the activity indices of SCD-18, D6D, and D5D were all statistically significant in predicting MetS 20 years later. Low activity indices of SCD-18 (OR = 0.22, *P* < 0.05) and D5D (OR = 0.18, *P* = 0.02), along with a high activity index of D6D (OR = 5.58, *P* < 0.01), significantly increased the risk of MetS at age 70. *In model 4*, the FAs including 16:1n-7, 18:0, 18:3n-3, 20:3n-6 and 20:5n-3 in CE of healthy men at age 50 were significant predictors of MetS 20 years later. High levels of 16:1n-7 (OR = 6.00, *P* = 0.01), 18:0 (OR = 6.74, *P* = 0.02), 20:3n-6 (OR = 24.3, *P* < 0.001), and 20:5n-3 (OR = 2.91, *P* = 0.01) significantly increased the risk of MetS, while low levels of 18:3n-3 (OR = 0.22, *P* = 0.05) were not a predictor of MetS at age 70. *In model 5*, both FBG (OR = 1.54, *P* = 0.01) and AST (OR = 28.3, *P* < 0.001) at age 50 significantly predicted MetS 20 years later. *In model 6*, high levels of TG (OR = 1.69, *P* < 0.001) and apoB (OR = 5.87, *P* < 0.001) significantly increased the risk of MetS, along with low levels of HDL-C (OR = 0.44, *P* = 0.004) at age 70. Additionally in *model 7*, high levels of DBP (OR = 1.03, *P* < 0.01) at age 50 significantly increased the risk of MetS at age 70.

### MetS prediction model at the age of 70 (Model 8)

The final combined model is a multivariate binary logistic regression model considering BMI-related SNPs, clinical metabolic risk parameters, and FA and lipoprotein-related variables at age 50. As shown in Table 6, the model is a strong predictor of MetS risk at age 70. The risk of developing MetS at age 70 was found to be significantly increased in BDNF rs7103411 heterozygotes (OR = 1.95, *P* = 0.01), and homozygotes for the minor allele of FTO rs1558902 (OR = 3.07, *P* = 0.003), with low activity of D5D (OR = 0.05, *P* = 0.02), high levels of 20:5n-3 (OR = 15.5, *P* < 0.001), FBG (OR = 1.84, *P* = 0.01), and AST (OR = 17.7, *P* < 0.001), low levels of HDL-C (OR = 0.16, *P* < 0.001), and high levels of apoB (OR = 10.2, *P* < 0.001).

**Fig. 2:**
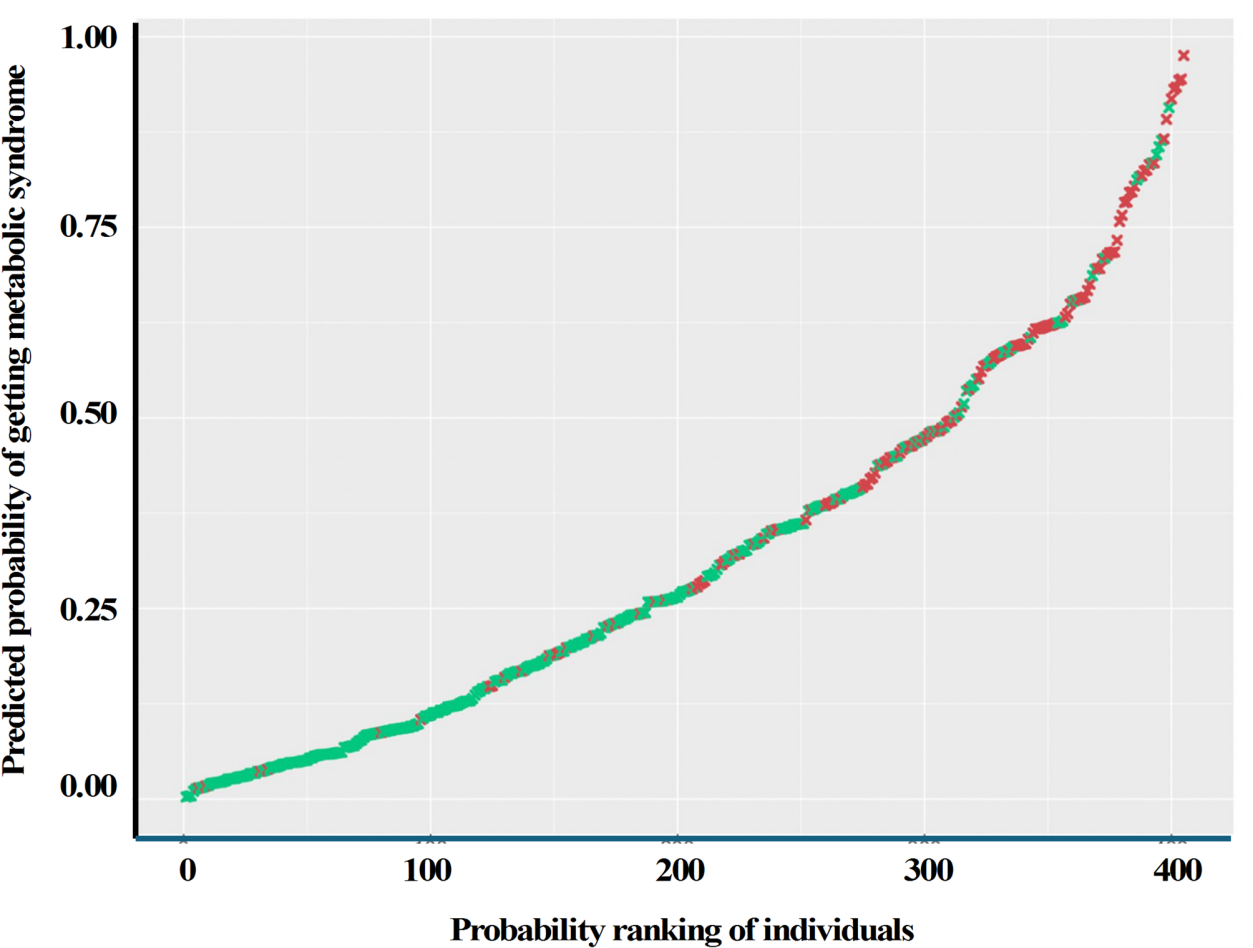
Predicted probabilities of each individual in the ULSAM cohort having metabolic syndrome (MetS) at age 70 along with their actual MetS status at age 70. The combined model 8 (Table 6) was used to calculate the probability of each subject having the outcome of MetS at age 70, and the 407 individuals that had no missing variables (Fig. 1) ranked in order from lowest to highest probability. The X-axis shows the ranking of each individual for the probability of having MetS, and the Y-axis shows, on a scale from 0 to 1, the predicted probability for each subject for developing MetS at age 70. Green x´s represent the negative outcomes for MetS, and red x´s represent the positive outcomes for MetS

Figure 2 illustrates the performance of the combined statistical model 8, by showing prediction probabilities of MetS among each individual in the ULSAM cohort (age 50) at age 70 along with their actual MetS status at age 70. The x-axis demonstrates probability of developing MetS at the age of 70, ranked in order from lowest to the highest including 407 individuals (Fig. 1) with no missing variables. The y-axis demonstrates the predicted probability for each subject for developing MetS at age 70. Most of the 132 individuals who developed MetS (Fig. 1) within 20 years are predicted to have a high probability of having MetS, and most of the 275 individuals who did not develop MetS (Fig. 1) are predicted to have a low probability of MetS.

## Discussion

We demonstrate for the first time that BMI-related SNPs are associated with FA profiles in serum CE and lipid metabolism, and contribute to the long-term risk of MetS in older age. Several BMI-related SNPs were linked to D5D enzyme activity and serum HDL-C levels at age 50 in individuals from the large Swedish cohort of men (ULSAM). Notably, two SNPs—*BDNF* (rs7103411) and *FTO* (rs1558902)—predicted the development of MetS at age 70 in men who did not have MetS at age 50. Our combined model at age 50 identified BDNF (rs7103411) and FTO (rs1558902) variants, along with metabolic parameters such as low D5D enzyme activity, high levels of 20:5n-3 in serum CE, FBG, AST, apoB, and low serum HDL-C levels, as significant risk factors for MetS at age 70 in men.

The BDNF gene was recently shown to be involved in obesity-related pathways in obese children [21]. BDNF encodes a growth factor with best-characterized roles in the CNS and regulates many target genes [22]. BDNF protein is the most abundant neurotrophin in the brain and essential for neuronal survival during development and integrating neurons in the adult brain [23]. Furthermore, evidence from studies in humans and rodents indicate that BDNF signalling plays a key role in regulating feeding, energy expenditure, and glycemic control [24]. Importantly, BDNF controls the appetite through a combination of central and peripheral pathways, and it is widely expressed in the fed state but suppressed after food deprivation [25]. BDNF and FTO are shown to be co-regulated in murine hypothalamic cell types [26] and may thus influence similar RNA modifications. We observed an increased risk of MetS at age 70 for carriers of the rare alleles at these two loci. For BDNF, the effect was most significant for heterozygote carriers.

The nucleic acid demethylation activity of the FTO enzyme could provide a mechanism through which the expression of genes involved in FA synthesis may be affected [27]. Recently, it was shown that hepatic lipid accumulation in chickens is mediated by FTO-dependent m6A demethylation of lipogenic mRNAs involved in FA metabolism [28]. BDNF rs7103411 and FTO rs1558902 most likely induce RNA modifications, as FTO is co-regulated with BDNF [26] and regulates many target genes [29]. This is in line with our findings, where both BDNF rs7103411 and FTO rs1558902 were significantly associated with D5D activity, and all three variables were significant risk predictors of MetS at age 70.

High D5D activity is known to be protective against MetS [5]. When investigating the ULSAM cohort, Warensjö et al. [6] suggested that the association of MetS with D5D enzyme activity has a genetic link, as it was independent of lifestyle factors and inversely associated with MetS risk. The present study supports this hypothesis, as D5D activity had the strongest associations with BMI-linked SNPs. Therefore, it was also used as a predictor in the final statistical model, where it was a significant predictor of MetS. This finding suggests a possible functional role of BDNF rs7103411 and FTO rs1558902 in the development of MetS, which may be mediated through modifications of genes regulating hepatic D5D activity.

We also discuss the association of one locus with obesity, even though it did not persist in the final model. The ETV5 gene is involved in hepatic FA metabolism by binding to PPAR response elements, and the ETV5 transcription factor has been associated with obesity in genomic association studies [30, 31, 32]. This is consistent with our results, where homozygotes for the minor allele of ETV5 rs9816226 were at a significantly higher risk for MetS. We show for the first time that ETV5 rs9816226 reached a significant Benjamini-Hochberg corrected P-value with HDL-C at both ages 50 and 70. This may indicate a dysregulation in HDL-C, which may in turn play a role in the development of MetS later in life.

A high level of apoB is a well-known risk factor for MetS [31, 33]. A recent study has demonstrated that apoB predicts the long-term prognosis for coronary atherosclerosis to a considerable extent in patients with diabetes, obesity, and MetS [34]. Our study confirmed that apoB is an important predictor of MetS.

The endogenous synthesis of the marine-derived n-3 PUFAs 20:5n-3 occurs mainly in the human liver and is catalyzed by the D5D enzyme. Several studies have indicated associations between endogenous FA synthesis and genetic variations in the D5D-encoding gene FADS1 [35, 36, 37]. The anti-inflammatory and hypotriglyceridemic properties of the n-3 PUFA 20:5n-3 are well known [38]. A recent systematic review and meta-analysis [39] suggest that consuming marine-derived n-3 PUFAs through diet or supplementation reduces serum pro-inflammatory eicosanoid synthesis in obese and overweight individuals. This may be explained by differences in the genetic background and dietary patterns of different study populations. For example, 20:5n-3 has been suggested to be modulated by genetics, as decreased anti-inflammatory response to 20:5n-3 was shown in individuals with a high genetic risk score for predisposition to low-grade inflammation associated with obesity [40]. It was previously demonstrated in individuals of the present study that the 20:5n-3 levels in serum CE did not differ between individuals who developed MetS at age 70 and those who did not [6]. Contrary to expectations, our multifactor model indicates that a relatively high 20:5n-3 level in serum CE is a risk factor for MetS. While supplementation with 20:5n-3 for the improvement of obesity and MetS in humans has not consistently been proven to be beneficial, results suggest it may improve the metabolic profile [41].

The strengths of our study lie in its longitudinal design, large sample size, and homogeneity of the individuals. The present study provides unique insight into predictive factors of MetS, as it considers genotypes along with intermediate phenotypes using lipid and FA metabolism at age 50 to predict outcome phenotypes of MetS status at age 70. However, the study was not without limitations. The less conservative approach of Benjamini-Hochberg correction was used to account for false positives with relaxed causality assumptions, as many of the SNPs with possible causal effects were in LD and are thus, by definition, not independent. Discarding some of them may have only captured a small proportion of trait variance explained, and it was not considered beneficial, as the analysis was used as a funnelling tool for further MetS risk assessment of candidate SNPs in binary regression models. In addition, the FA percentages and the desaturases are not independent as they are proportions derived from the total amount of FAs in CE. Therefore, an increase in the proportion of false negative findings might have been caused by a Bonferroni correction, which was consequently considered conservative for this study. Furthermore, the study on ULSAM cohort exclusively consisted of Swedish men, limiting the generalizability to women and other ethnicities. Replicate analyses in other cohorts of more general populations consisting, e.g., of mixed ethnicities and comprising both sexes are recommended to confirm generalizability of these associations. It should be noted that the visual representation of predicted probabilities for MetS using the final additive model (Model 8) in the ULSAM cohort (Fig. 2) was generated using the same dataset on which the model was trained. As no independent test set or cross-validation was applied, the results may reflect characteristics specific to this dataset and should be interpreted with caution. Future studies should incorporate internal validation methods, such as cross-validation, or use external datasets to evaluate the model’s predictive performance more rigorously. While the combined use of metabolic and genetic variables is a strength and novelty of the study, we acknowledge that the theoretical possibility of overfitting remains, as the final model includes multiple covariates. Further analyses of genome-wide genetic variations of all types, along with multivariate analyses or the application of machine learning methods to the combined genotype and phenotype matrix, could potentially reveal additional insights into associations between predictor variables and the risk of MetS. While these analyses cannot prove a cause-effect relationship, they do provide a hypothesis upon which future studies can be based.

## Conclusions

This study provides novel evidence linking BMI-related SNPs to FA metabolism and lipoproteins. It demonstrates that genetic markers and their associated lipid-related markers strongly predict the long-term risk of MetS in older age. Specifically, genetic variants in *FTO* and *BDNF*, along with critical metabolic variables including serum levels of 20:5n-3, FBG, AST, apoB, and HDL-C at age 50, were identified as significant predictors of MetS at age 70. These findings highlight the strong interplay between genetic predisposition and dysregulation in FA and lipid metabolism in the development of MetS. Further research is needed to elucidate the exact mechanisms underlying these associations. The insights from this study pave the way for improving predictive models for MetS and developing targeted interventions to mitigate MetS risk.

## Electronic supplementary material

Below is the link to the electronic supplementary material.

Supplementary Material 1

## Data Availability

Data is provided within the manuscript and supplementary information files.

## Abbreviations

MetS: Metabolic syndrome
SNPs: Single-nucleotide polymorphisms
FA: Fatty acid
ULSAM: Uppsala Longitudinal Study of Adult Men
BMI: Body mass index
BDNF: Brain-derived neurotrophic factor
FTO: Fat mass and obesity-associated gene
D5D: Delta-5-desaturase
D6D: Delta-6-desaturase
CE: Cholesteryl esters
SCD: Stearoyl-CoA desaturase
HDL-C: High-density lipoprotein cholesterol
TG: Triglycerides
LDL-C: Low-density lipoprotein cholesterol
TC: Total cholesterol
AST: Abdominal skinfold thickness
ApoB: Apolipoprotein B
WC: Waist circumference
SBP: Systolic blood pressure
DBP: Diastolic blood pressure
FBG: Fasting blood glucose
