# Supplementary material for: The interplay of genetics and fatty acid metabolism: exploring their impact on metabolic syndrome in Swedish men
Source: Nutr J. 2025 Jul 1;24:99. doi: 10.1186/s12937-025-01168-8 (PMC12210471; doi:10.1186/s12937-025-01168-8)
Supplement: Supplementary file 1 — Supplementary Material 1 [file 12937_2025_1168_MOESM1_ESM.docx]

**The interplay of Genetics and Fatty Acid Metabolism: Exploring their impact on Metabolic Syndrome in Swedish Men**

Harpa Oskarsdottir^2^, Arnar Palsson^3^, Erla B. Olafsdottir^3^, Salahuddin Mohammad^1^, Ulf Risérus^4^, Helgi B. Schiöth^1^, Gudrun V. Skuladottir^1,2#^, Jessica Mwinyi^1#^*

**Supplementary Material:**

**Table S1.** SNP IDs of proxies were used for some of the selected SNPs.

**Table S2a.** Significant associations between SNPs and fatty acid metabolizing enzymes, serum cholesteryl ester (CE) fatty acids, and HDL-C of the ULSAM cohort at age 50.

**Figure S1.** Schematic representation of the 68 SNPs, including all proxies and the eight different LD blocks.

**Figure S2.** Correlation plot between all fatty acids in serum cholesteryl ester and activity indices of their desaturases at age 50.

**Figure S3.** Correlation plot between variables of ULSAM at age 50.

**Table S3**. Baseline characteristics of participants (at age 50) lost during follow-up at age 70 in the ULSAM cohort, *N* = 1101

| 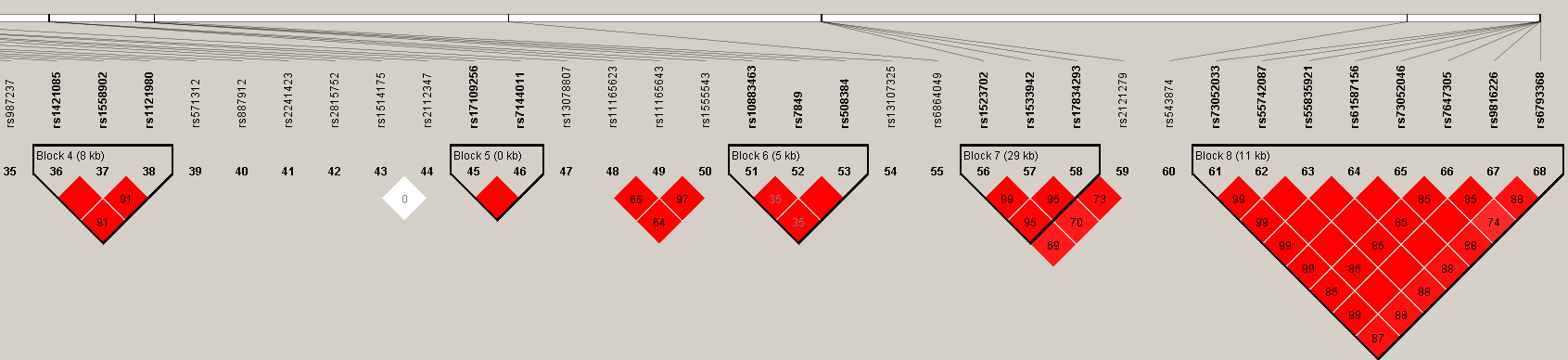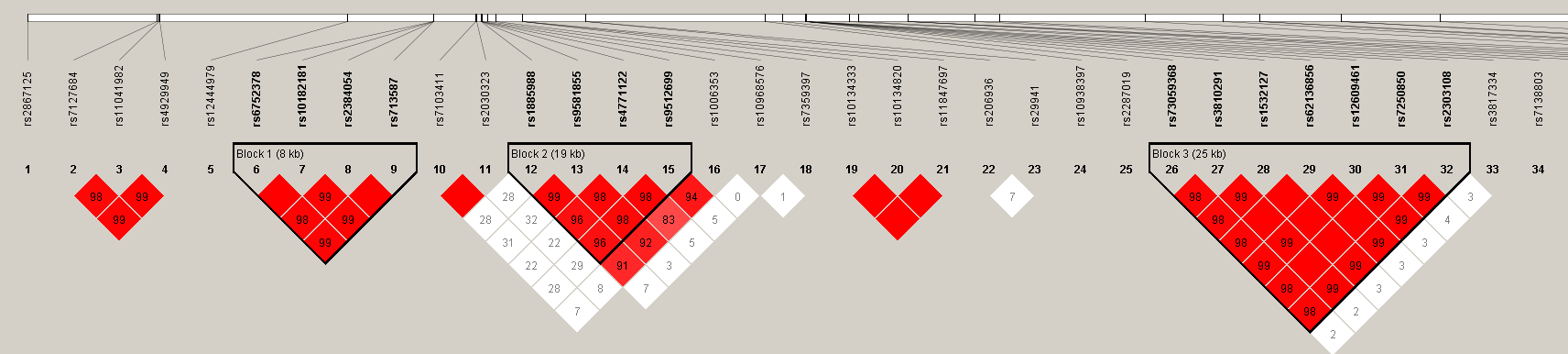 |
| --- |
| ***Figure S1****. Schematic representation of the 68 SNPs, including all proxies and the eight different LD blocks. The LD is expressed as R^2^ between the pairs of 68 SNPs genotyped in the ULSAM cohort. R^2^ values are given in percentages (from 0 to 100), where high values are in red and low values are in white. The haplotype analysis was performed using Haploview 4.2.* |

| 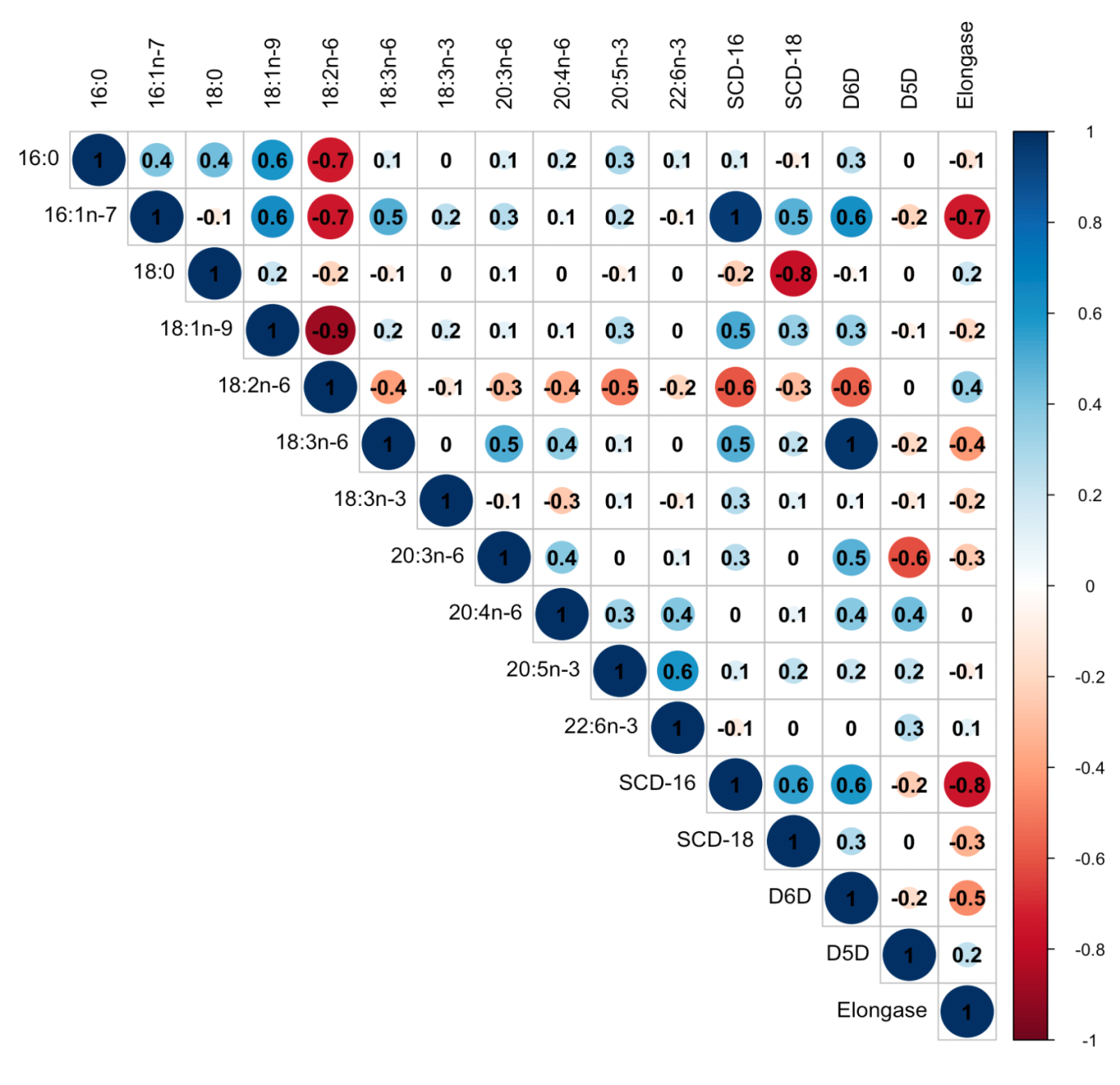 |
| --- |
| ***Figure S2.*** *Correlation plot between all fatty acids in cholesterol ester and activity indices of their desaturases and elongase at age 50. The correlation coefficients are represented as numbers (rounded to one decimal place), and the type of correlation is indicated in blue (positive) or in red (negative).* |

| 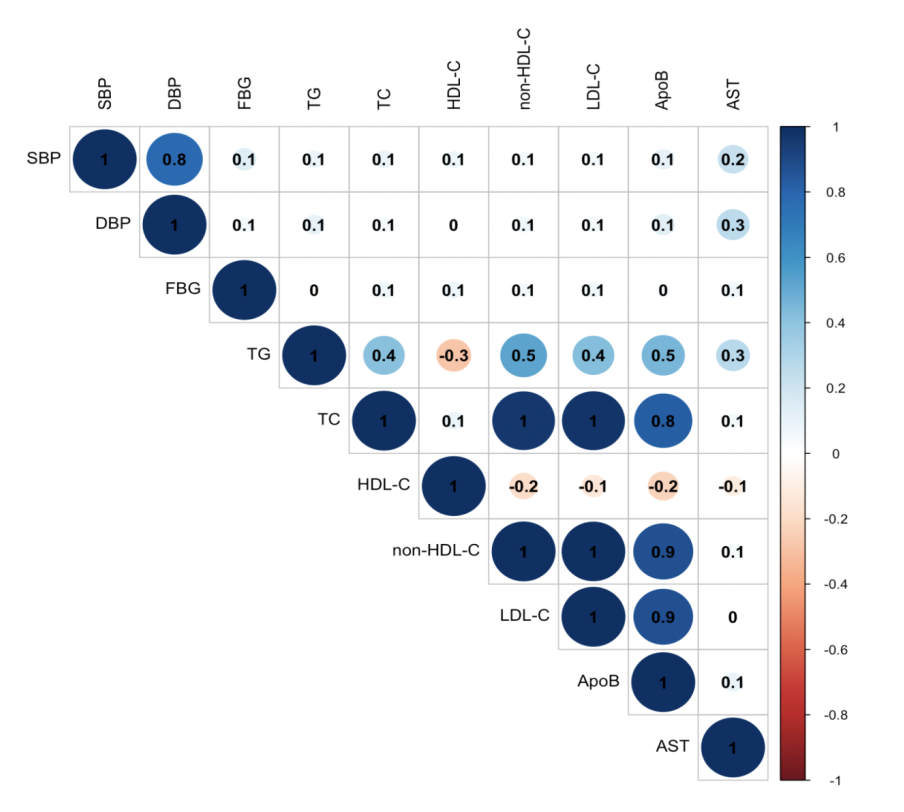 |
| --- |
| ***Figure S3.*** *Correlation plot between variables of ULSAM at age 50. The correlation coefficients are represented as numbers (rounded to one decimal place), and the type of correlation is indicated in blue (positive) or in red (negative).* |

| **Table S3**. Baseline characteristics of participants (at age 50) lost during follow-up at age 70 in the ULSAM cohort, *N* = 1101 | | | |
| --- | --- | --- | --- |
|  | **Median** | **(Max, Min)** | **Missing, *N*** |
| **BMI** (Kg/m2) | 24.91 | (42.12, 16.93) | 1 |
| **AST** (mm) | 18.4 | (50, 4.2) | 464 |
| **WC** (cm) | 89 | (122, 71) | 869 |
| **SBP** (mmHg) | 130 | (235, 100) | 2 |
| **DBP** (mmHg) | 85 | (145, 50) | 2 |
| **FBG** (mmol/L) | 4.89 | (16.17, 3.19) | 5 |
| **TC** (mmol/L) | 6.79 | (13.8, 2.81 | 1 |
| **HDL-C** (mmol/L) | 1.28 | (4.32, 0.34 | 210 |
| **LDL-C** (mmol/L) | 5.24 | (12.25, 2.05) | 210 |
| **Non-HDL-C** (mmol/L) | 5.59 | (13.02, 2.05) | 209 |
| **TG** (mmol/L) | 1.75 | (24.68, 0.56) | 1 |
| **ApoB** (g/L) | 1.24 | (2.34, 0.46 | 241 |
| *Data presented as Median and Range (Max, Min); Missing, N, number of missing participant data. BMI, body mass index; AST, abdominal skinfold thickness; WC, waist circumference; SBP, systolic blood pressure; SBP, diastolic blood pressure; FBG, fasting blood glucose; TC, total cholesterol; TG, triglyceride; ApoB, apolipoprotein B.* | | | |
